# Cryo-EM insight into the structure of MTOR complex 1 and its interactions with Rheb and substrates

**DOI:** 10.12688/f1000research.16109.1

**Published:** 2019-01-03

**Authors:** Luke H. Chao, Joseph Avruch

**Affiliations:** 1 Department of Molecular Biology, Massachusetts General Hospital, Boston, MA, USA; 2 Department of Genetics, Harvard Medical School, Boston, MA, USA; 3 Department of Medicine, Harvard Medical School, Boston, MA, USA

**Keywords:** MTOR, Raptor, mLst8, PRAS40, AKT1S1, Rheb, p70 S6 kinase, S6K1B, EIF4EBP1, 4E-BP

## Abstract

The mechanistic target of rapamycin (MTOR) is a giant protein kinase that, together with the accessory proteins Raptor and mLst8, forms a complex of over 1 MDa known as MTOR complex 1 (MTORC1). MTORC1, through its protein kinase activity, controls the accretion of cell mass through the regulation of gene transcription, mRNA translation, and protein turnover. MTORC1 is activated in an interdependent manner by insulin/growth factors and nutrients, especially amino acids, and is inhibited by stressors such as hypoxia and by the drug rapamycin. The action of insulin/growth factors converges on the small GTPase Rheb, which binds directly to the MTOR polypeptide in MTORC1 and, in its GTP-bound state, initiates kinase activation. Biochemical studies established that MTORC1 exists as a dimer of the MTOR/Raptor/mLst8 trimer, and progressive refinements in cryo-electron microscopy (cryo-EM) have enabled an increasingly clear picture of the architecture of MTORC1, culminating in a deep understanding of how MTORC1 interacts with and phosphorylates its best-known substrates—the eIF-4E binding protein/4E-BP, the p70 S6 kinase/S6K1B, and PRAS40/AKT1S1—and how this is inhibited by rapamycin. Most recently, Rheb-GTP has been shown to bind to MTORC1 in a cooperative manner at an allosteric site remote from the kinase domain that twists the latter into a catalytically competent configuration. Herein, we review the recent cryo-EM and associated biochemical studies of MTORC1 and seek to integrate these new results with the known physiology of MTORC1 regulation and signaling.

## Introduction

The giant protein kinase mechanistic target of rapamycin (MTOR) operates in two physically distinct and independently regulated multi-protein complexes called MTOR complex 1 (MTORC1) and MTOR complex 2 (MTORC2) ^1^. MTORC1, composed of the MTOR protein kinase and the non-catalytic polypeptides Raptor and mLst8 ^2, 3^, is a central regulator of cell function through its broad control of anabolic and catabolic processes ^4, 5^. TORC1 controls polypeptide abundance through the regulation of gene transcription by all three RNA polymerases ^6^, cap-dependent mRNA translation ^7^, and protein degradation via autophagy and the proteasome ^8^. Advances in structure determination—in particular, cryo-electron microscopy (cryo-EM)—have enabled considerable progress in understanding the structure of both MTORC1 ^9– 14^ and MTORC2 ^15– 18^. Here, we review the current understanding of MTORC1 structure and its implications for the regulation and signaling by this important protein kinase.

## MTORC1 composition and architecture

**Figure 1.**
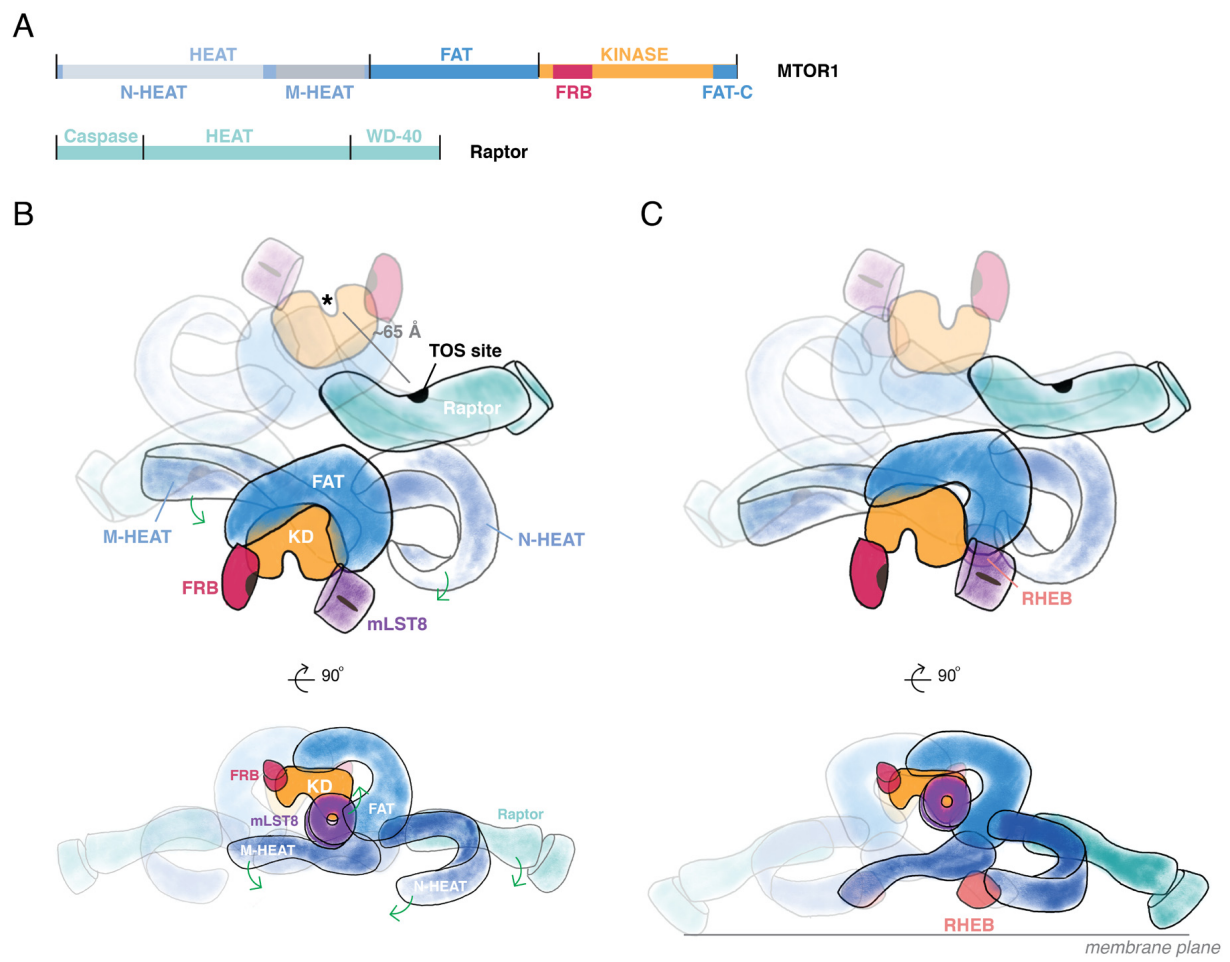
TOR complex 1: MTOR and Raptor domain structure and overall architecture with and without Rheb. ( **A**) Domain architecture of mechanistic target of rapamycin 1 (MTOR1). At the amino-terminus of MTOR, a set of HEAT repeats organize into the N- and M-HEAT domains. Following the HEAT region is the FAT (FRAP/ATM/TRRAP) domain, which precedes the catalytic kinase domain. The kinase domain includes the FRB insertion (site of FKBP12:rapamycin binding) in the amino-terminal lobe and the FATC segment integrated into the carboxy-terminal lobe. Raptor contains an amino-terminal, catalytically inactive Caspase-like domain and a set of HEAT repeats followed by a beta-propeller formed by WD40 repeats. ( **B**) The MTOR complex 1 (MTORC1) holoenzyme arrangement. The dimeric MTORC1 assembles through interactions between one M-HEAT domain and its partner’s N-HEAT. Raptor interacts near this interface. Substrate recognition sites—for example, the TOR signaling (TOS) site—are marked in black. The HEAT domains project from the FAT domain. The FAT domain wrap of the kinase domains projects the active sites away from one another and the central twofold axis. ( **C**) Conformational rearrangement of MTORC1 induced by Rheb-GTP. Membrane-localized Rheb-GTP binds to a site formed by the intersection of FAT and M- and N-HEAT domains. Formation of this site induces repositioning of N- and M-HEAT domains and, as a result, Raptor. Repositioning of N- and M-HEAT domains propagates conformational change through the FAT domain to influence catalytic activity of the kinase domain. If MTORC1 interacts with two membrane-localized Rheb-GTP molecules, MTORC1 would be predicted to sit with the top view orthogonal to the membrane plane.

MTOR is a member of the phosphatidylinositol 3-kinase (PI3K)-related kinases (PIKKs), along with ATM, ATR, DNA-PK, SMG1, and TRRAP (a pseudokinase), which share a catalytic domain that more closely resembles that of the phosphatidylinositol 3′ (lipid) kinases than that of the typical eukaryotic protein kinases ^19, 20^. The amino-terminal 1,345 residues of the approximately 289 kDa MTOR polypeptide contain 32 HEAT repeats, each a 30- to 40-residue segment consisting of two antiparallel alpha-helices connected by a flexible linker ^21, 22^. The HEAT domains can be further subdivided into the N- and M-HEAT domains ^12^. This is succeeded by the roughly 500-residue FAT (FRAP/ATM/TRRAP) domain, followed by the MTOR catalytic domain ( Figure 1A).

The MTOR catalytic domain has a canonical two-lobed structure with key insertions in both the amino-terminal and the carboxy-terminal lobes. In the amino-terminal lobe, a long alpha-helix packs against the amino-terminal lobe, as seen in other PI3K family members. Following this helix is the FRB insertion, the site for FKBP12:rapamycin binding. In the carboxy-terminal lobe, a conserved, roughly 35-residue segment, FATC, seen only in the PIKKs that contain a FAT domain, stabilizes the activation loop and is completely integrated into the carboxy-terminal domain. Another segment in the carboxy-terminal lobe, referred to as LBE, forms a site for mLst8 binding. An insertion in the carboxy-terminal lobe plugs a pocket beneath helix alpha D (in the PKA nomenclature), a surface often used for substrate binding, leaving little room immediately proximal to the phosphorylation site for the form of sequence recognition interactions seen in protein kinases like PKA. The FRB domain extends over the catalytic cleft from the amino-terminal lobe, as do the FATC and LBE from the carboxy-terminal lobe, thereby creating a deeply recessed catalytic cleft. Modeling the FKBP12:rapamycin complex onto the FRB segment indicates that the latter severely limits access to the catalytic cleft. Moreover, the side of the FRB facing the catalytic cleft serves as a secondary substrate-binding site ^10, 14^; thus, inhibition by the FKBP12:rapamycin complex is not allosteric, as was long thought, but occurs by interference with substrate recruitment ^10, 11, 14^. The 40 kDa mLst8/Gβl polypeptide is composed entirely of seven WD40 repeats in a beta propeller; despite its status as a core component of MTORC1, its function in the complex is unknown inasmuch as its deletion does not alter MTORC1 signaling ^23^.

Crystal structures and cryo-EM reconstructions show that the FAT domain is a large open solenoid that envelops, through extensive interactions, the “back side” of the kinase domain (that is, the surface opposite that containing the catalytic cleft) ^12– 14^. MTORC1 operates as a dimer of the MTOR, mLst8, and Raptor heterotrimer ^9^, mediated by an antiparallel arrangement of the MTOR HEAT domains, which extend from the FAT alpha solenoid ^11– 14^ ( Figure 1B and 1C). The N-HEAT (also referred to as the “spiral” or “horn”) of one MTOR interacts with the M-HEAT (“bridge”) of the other MTOR polypeptide. This composite N-HEAT/M-HEAT surface provides a platform for Raptor. Raptor is a roughly 150 kDa polypeptide ^2, 3^ that contains an N-terminal, catalytically inactive ^24^ caspase-like domain, followed by HEAT repeats forming an open solenoid and seven WD40 repeats forming a closed solenoid known as a beta-propeller. Through the N-HEAT/M-HEAT interface, each Raptor engages both MTOR molecules; Raptor binding stabilizes but is not required to maintain the dimer. Raptor serves as the indispensable binding subunit for the canonical TORC1 substrates p70 S6 kinase/S6KB1 and EIF-4E binding protein/4E-BP ^3^; the dimer interface orients Raptor such that the N-terminal caspase-like domain, which mediates Raptor’s substrate-binding function, is positioned opposite to a kinase catalytic cleft ^11– 14^. The symmetric N-HEAT/M-HEAT interface also cooperatively couples the two Rheb sites, whereby binding to one site is expected to pre-configure the second site for higher-affinity binding ^14^.

## MTORC1 regulation

MTORC1 serves as a positive regulator of cell mass and proliferative capability in all eukaryotic cells, and much effort has been directed toward elucidating the regulation of TORC1 activity. In single-cell eukaryotes, TORC1 is activated primarily by nutrients, which are both the substrate and the stimulus for cell growth and proliferation. In metazoans, MTORC1 is activated by growth factors and cytokines and in an interdependent manner by nutrients, especially amino acids (AAs) ^4^. The model generally proposed for TORC1 activation in mammalian systems envisions two parallel pathways that converge at the lysosome. Growth factors and cytokines promote the activation of MTORC1 by inhibiting the tuberous sclerosis complex, a complex of TSC1, TSC2, and TBC1D17 that is the GTPase activator protein (GAP) for the ras-like small GTPase Rheb ^25^. Rheb binds directly to the MTOR polypeptide and, in its GTP-bound form, is required for TORC1 (but not TORC2) activation ^26^. The kinases controlled by PI3K (Akt) and Ras-GTP (MAPK1, Rsk) phosphorylate TSC2 and the Iκb kinases phosphorylate TSC1 so as to impair, by various mechanisms, TSC GAP function and thereby promote Rheb GTP charging ^25^. AAs act through an independent pathway that converges on the Rag GTPase heterodimer ^27, 28^, which in its activated state binds to the Raptor subunit of TORC1 and conveys TORC1 to the cytoplasmic surface of the lysosome ^28, 29^; there, MTOR is activated by interaction with Rheb-GTP. Withdrawal of AAs, especially leucine and arginine, markedly inhibits MTORC1 signaling despite maximal growth factor signaling ^30^. Conversely, overexpression of Rheb, which swamps endogenous TSC GAP activity, enables MTORC1 activation even in the absence of all AAs ^31^. Thus, a critical step in MTORC1 activation by insulin, growth factors, and AAs is the binding of Rheb-GTP to the MTOR polypeptide in MTORC1 at the surface of the lysosome ^4, 32^.

Multiple variants and exceptions to this model have been described; for example, in mammalian cells, whereas Leu acts via Rag recruitment of TORC1 to the lysosome, Gln has been observed to recruit TORC1 to the lysosome through Arf1 in a Rag-independent manner ^33^. Thomas *et al*. ^34^ described the ability of Rab1-GTP to promote TORC1 activation at the Golgi in a Rheb-dependent manner. In *Caenorhabditis elegans*, the TSC is entirely lacking and CeTORC1 is regulated independently of the InsR/IGFR Daf2 ^35^ except for transcriptional regulation of CeRaptor/Daf15 abundance ^36^. In lower eukaryotes, whereas TORC1 activation in *Schizosaccharomyces pombe* is SpRheb dependent ^37^, TORC1 regulation in *Saccharomyces cerevisiae* is entirely independent of ScRheb and sustained activation does not require the Rag GTPase Gtr1/Gtr2 and requires Gln rather than Leu; nevertheless, activation occurs at the vacuolar surface ^38^. *S. cerevisiae* contains about 200 TORC1 dimers per cell, distributed diffusely around the cytosolic face of the vacuole. Upon removal of extracellular glucose (but not AAs or ammonium), TORC1, while remaining associated with the vacuole, undergoes a rapid inactivation, accompanied by a Gtr1/Gtr2-mediated reorganization into a large hollow cylinder composed of 100 or more dimers ^39^. The packing of the TORC1 dimers in these assemblies is such that the central Raptor HEAT/armadillo repeat domain is apposed to the TOR FRB segment in a manner that resembles the binding of the inhibitory FKBP12:rapamycin complex; in fact, rapamycin interferes with the assembly of the cylinders. Based on these and other features, these cylindrical TORC1 assemblies have been designated “TOR organized in inhibited domains” or TOROIDs. Although the diverse mechanisms of TORC1 regulation ultimately will each require elucidation, here we focus on MTORC1 interaction with Rheb and with the preferred substrate PRAS40.

## MTORC1 signaling

The MTORC1 signaling output, as defined by its Ser/Thr kinase catalytic function, displays several unusual features. First, the presence of a substrate-binding subunit (that is, Raptor) separate from the kinase polypeptide itself is distinctly uncommon. Raptor binds the substrates S6K1B and 4E-BP through their TOS (TOR signaling) motif (F/Ac/ϕ/Ac/ϕ; Ac = acidic, ϕ = hydrophobic) ^40^; the Raptor binding motif in PRAS40 is slightly variant (FVMDE) ^41, 42^. The integrity of their TOS motifs and the binding of these substrates to Raptor are necessary for their phosphorylation by MTORC1, both *in vitro* and in the cell ^43– 45^. However, at least one physiologic MTORC1 substrate, the Igf2 mRNA-binding protein, IGF2BP2/IMP2, does not bind Raptor but binds to MTOR directly and is phosphorylated by MTORC1 *in vivo* and *in vitro* independently of Raptor ^46^. A systematic analysis of the increasing number of MTORC1 substrates for the Raptor dependence of their phosphorylation is awaited. A second unusual feature of MTORC1 is that its phosphorylation site selection is relatively broad; all Ser/Thr sites on 4E-BP, on the carboxy-terminal non-catalytic tail of S6K1B, and on IMP2 are followed immediately by a Pro residue, whereas the critical regulatory MTORC1 phosphorylation site on S6K1B, Thr389/412, is situated in the highly hydrophobic motif FLGF TYVA. Surprisingly, one report describes the ability of MTORC2 to catalyze tyrosine phosphorylation of the insulin and IGF1 receptors ^47^.

The PRAS40 polypeptide is multiply phosphorylated by MTORC1 ^48^. PRAS40 binds strongly to Raptor and, in contrast to the canonical physiologic TORC1 substrates 4E-BP or S6K1B, is commonly retrieved in stable association with MTORC1. Overexpression of PRAS40 strongly inhibits the phosphorylation of both 4E-BP and S6K1 ^41, 42, 49, 50^, which themselves show cross-competition for phosphorylation by MTORC1 ^51^, suggesting that access to Raptor may be limiting for substrate phosphorylation ^52^. PRAS40 remains bound to Raptor despite its MTOR-catalyzed phosphorylation unless it is also phosphorylated by Akt at a carboxy-terminal site, Thr246, which enables its binding to 14-3-3 and release from Raptor ^42, 49, 50^. This behavior, together with the usual residency of PRAS40 in the unstimulated MTORC1 complex, led to the proposal that PRAS40 may serve as an Akt controlled regulator of substrate access to MTORC1 ^42, 49, 50^, acting in concert with Akt inhibition of TSC and activation of Rheb to promote optimal MTORC1 activation. However, support for this plausible and attractive hypothesis is scant; despite its ubiquitous expression and in contrast to inactivation of TSC1 or TSC2, inactivation of the *AKT1S1/PRAS40* gene in mice ^53, 54^ or Drosophila ^55^ does not result in global phenotypes indicative of enhanced MTORC1 signaling, although tissue-specific effects are described ^54, 55^. Depletion of PRAS40 in cell culture has been reported to promote ^42, 49, 50^, impair ^56, 57^, or not alter ^58^ insulin/growth factor-stimulated MTORC1 activity. The robust activation of MTORC1 by Rheb overexpression ^58^ or treatment with phorbol esters (via Ras-GTP) ^59^ occurs without any displacement of PRAS40 from MTORC1. Although its physiologic functions remain obscure, PRAS40 is clearly a preferred MTORC1 substrate and an excellent model for how MTOR recognizes Raptor-bound substrates.

Cryo-EM structures show three potential PRAS40 interaction sites in MTORC1 ^14^: the PRAS40 TOS motif and the adjacent N-terminal residue binds to a pocket in Raptor formed by a cleft between the caspase-like domain and the succeeding solenoid structure. A set of arginine residues in the TOS binding pocket generates a positive electrostatic potential that favors acidic residues in flanking regions proximal to the consensus sequence. The site on Raptor occupied by the PRAS40 TOS motif is essentially superimposable on that occupied by the 4E-BP and S6K1 TOS motifs. Raptor localizes the TOS site about 65 Å from the active site of the kinase, increasing the local concentration of the substrate. PRAS40 also binds to two other sites within MTORC1. A PRAS40 long amphipathic alpha-helix (residues 212–232) makes an extensive interface with the FRB segment of the MTOR catalytic domain, positioning the substrate near the catalytic cleft; S6K1 and 4E-BP each use a region about 15 residues C-terminal to their phosphorylation sites to interact in a similar manner with the FRB segment, and mutation of the FRB-interacting sequences in S6K1 and 4E-BP markedly impairs their phosphorylation. In addition, PRAS40 interacts with a WD40 domain of mLST8 through a beta-strand interaction that is not observed with S6K1 or 4E-BP; this third site of PRAS40 interaction within the complex may account for the greater overall affinity for PRAS40 as compared with S6K1 and 4E-BP. How these interactions facilitate the processive phosphorylation of substrates by MTOR is unclear.

## Activation by Rheb

**Figure 2.**
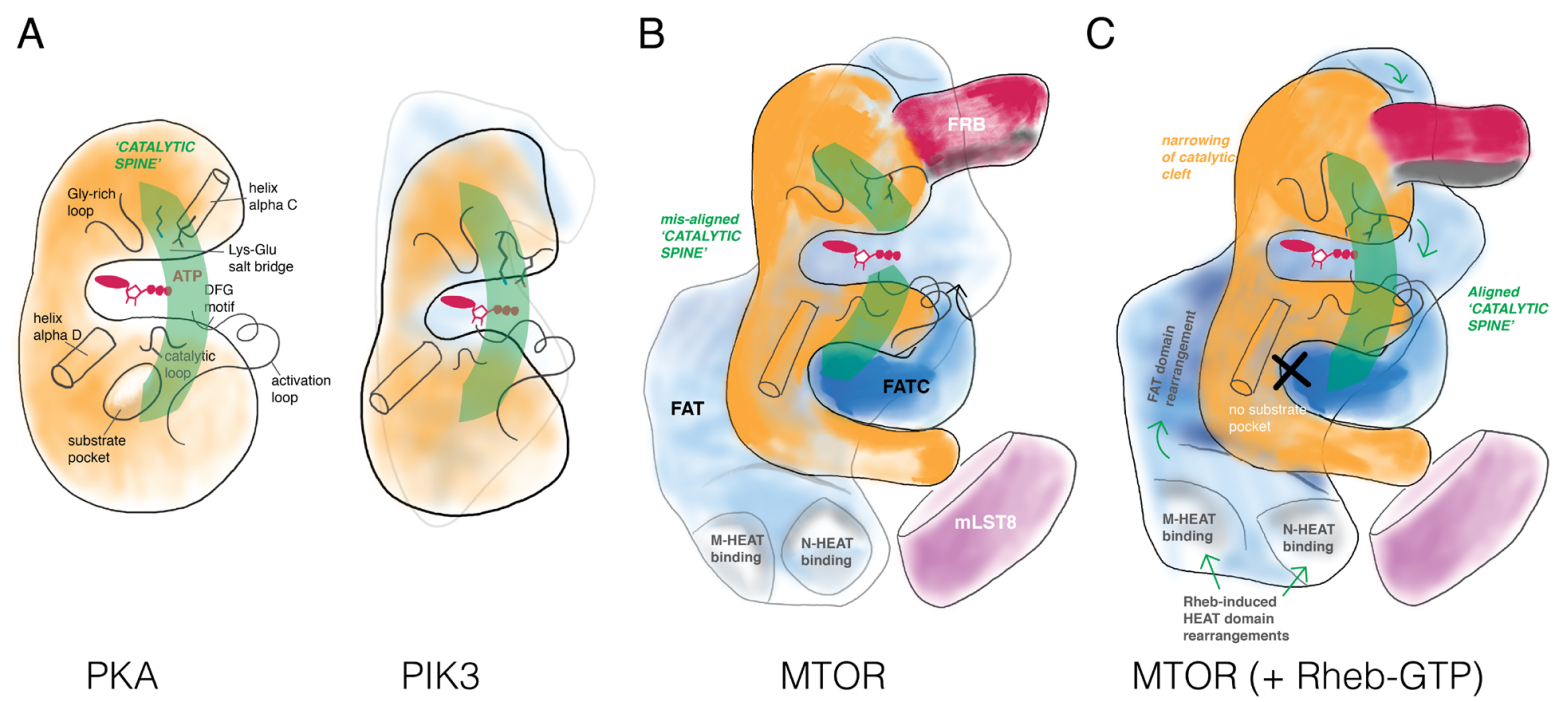
Kinase domain regulation. Several features define a catalytically active kinase domain: ( **A**) Protein kinase A (PKA) aligns key secondary structure elements for catalysis in a “catalytic spine”, which positions nucleotide (a Gly-rich loop forms a roof for the adenine base and a Lys-Glu salt bridge coordinates phosphate groups of ATP) and substrate (often through a pocket adjacent helix alpha D) for phospho-transfer (mediated by the catalytic loop and the DFG motif). Phosphatidylinositol 3-kinase (PI3K) shares these core catalytic elements with PKA. A defining element in PI3K is a series of tetratricopeptide (TRP) repeats that contacts the N-terminal and C-terminal lobes positioning the “catalytic spine” in register. ( **B**) Mechanistic target of rapamycin (MTOR) does not have a substrate-binding pocket next to the helix alpha D (explaining its rather broad substrate selectivity); instead, substrate docking elements distributed throughout the complex (for example, FRB) serve as the targeting/specificity determinants. In the context of the MTORC1 complex, the FAT domain of MTOR breaks the catalytic spine by locking the N- and C-lobes in a non-productive twisted arrangement relative to one another, resulting in a slightly wider catalytic cleft. ( **C**) Binding by Rheb-GTP to MTORC1 induces dramatic long-distance domain rearrangement of the N- and M-HEAT domains, resulting in a conformational change in the FAT domains, to align the catalytic spine in the kinase domain.

As demonstrated by Yang *et al*. ^14^, the catalytic domain in “apo” MTORC1 (that is, in the absence of Rheb) exhibits a wide catalytic cleft, prohibiting effective phosphate transfer from ATP bound at the P-site to polypeptide substrates. This conformation is enforced by the FAT domain, which clamps to both the amino- and carboxy-terminal lobes of the catalytic domain. The individual catalytic residues are positioned in a primed, active orientation; however, in contrast to, for example, PKA or PI3K ( Figure 2A), the FAT domain clamp twists the N- and C-lobes relative to one another, breaking the catalytic “spine” and widening the catalytic cleft such that the relative positions of residues in the two lobes are out of alignment in a conformation not conducive for catalysis ( Figure 2B). In this state, the N-heat and M-heat domains extend from the FAT domain to form a large open interface. The dimeric MTORC1 assembly is observed to undergo a “breathing” motion that couples the state of the two interfaces.

The cryo-EM reconstructions establish that Rheb-GTP binds to an interface composed of segments of the N-HEAT, M-HEAT, and FAT domains; the largest interaction surface is between Rheb and the N-HEAT which may initiate the overall interaction inasmuch as it is accompanied by a substantial displacement of the N-HEAT segment toward the interface where the M-HEAT and FAT domains meet ( Figure 1C). In this reconfigured state, the N-HEAT domain exhibits new interactions with the FAT domain, the middle portion of which undergoes a twist that loosens its interaction with the amino-terminal lobe of the catalytic domain. This allows the amino-terminal lobe to move closer to the carboxy-terminal lobe, closing the catalytic cleft substantially, accompanied by a marked increase in k _cat_, whereas K _m_ for the peptide substrate is little changed ( Figure 2). Some mutations in the FAT domain accompanied by constitutive activation of MTORC1 activity appear to mimic the effect of Rheb binding inasmuch as their kinase activity is not additive with maximal concentrations of Rheb ^14^. The minimal effect of Rheb on peptide affinity is at variance with that observed by Sato *et al*. ^60^, who found that Rheb-GTP caused a marked increase in the affinity of MTORC1 for 4E-BP. The switch I domain of Rheb, whose configuration is strongly altered upon GTP binding ^61^, exhibits interactions with the M-HEAT and FAT domains, whereas the switch II segment, whose configuration hardly differs whether Rheb is GTP- or GDP-bound, interacts with all three MTOR segments and is also critical to MTORC1 activation ^62^. The site of Rheb-MTOR interaction identified by cryo-EM, accompanied by extensive biochemical verification of Rheb-GTP activation of MTORC1, invalidates the earlier conclusion that Rheb binds to the MTOR catalytic domain, which was based on the use of Rheb co-expression with MTOR fragments ^26^. Rheb binding to MTOR *in vitro* exhibits positive cooperativity, and the unique configuration of MTORC1 bound to Rheb as seen in cryo-EM enabled the conclusion that MTORC1 dimers always show either two apo-MTORC1s or two Rheb-MTORC1s. The half-maximal concentration for Rheb activation of MTORC1 *in vitro* is about 100 μM, which is consistent with the very low affinity of the Rheb-MTOR interaction identified in early studies. Those studies showed that recombinant MTOR bound more tightly to mutant, nucleotide-free, or GDP-Rheb than to Rheb-GTP; however, the recombinant MTOR polypeptides retrieved with mutant, inactive Rheb were essentially devoid of catalytic activity *in vitro*. This finding was interpreted to indicate that MTOR interaction with Rheb-GTP was required to configure MTOR to an active state, a conclusion consistent with genetic data from Drosophila ^63, 64^ and with the recent results of Yang *et al*. ^14^. Nevertheless, the data thus far continue to leave unanswered the question of whether a Rheb-MTORC1 interaction is required to maintain MTORC1 in an active state.

Conceivably, there may be additional interactions or modifications (or both) of MTORC1 activity involved in Rheb activation, as is the case, for example, for the Ras-GTP regulation of the Raf kinases; the initial high-affinity Ras-GTP/Raf interaction ^65^ is followed by a series of post-translational modifications of Raf and altered protein-protein interactions that result in a stable, Ras-free, Raf active state ^66^. However, evidence is lacking for a stable activation of MTOR catalytic function (that is, k _cat_) in MTORC1 in response to cell stimulation. Unlike many conventional protein kinases, the MTOR activation loop is not subject to phosphorylation and the orientation of the critical catalytic segments of the MTOR kinase domain in apo-MTORC1 is already in an active configuration, requiring only the Rheb-induced narrowing of the catalytic cleft to promote kinase activity. Reports detecting insulin activation of the kinase activity of MTORC1 assayed *in vitro* appear to reflect increased access of the added Raptor-dependent substrate (4E-BP or S6K1) ^49, 52^; no differences are evident using a Raptor-independent substrate or salt washes to deplete PRAS40 from the complexes ^58^. It has not been possible to retrieve endogenous MTOR in complex with endogenous Rheb after cell disruption (although a stable complex of SpTor2 with a mutant hyperactive SpRhb1 (K120R) has been observed ^35^). It can be argued that the very weak affinity of Rheb for MTOR results in a loss of the Rheb-MTOR interaction upon cell extraction; however, this returns to the question of how the Rheb activation mechanism demonstrated by Yang *et al*. ^14^ relates to the mechanism operative in the cell and whether a continuing association of Rheb with MTOR is required for the maintenance of MTORC1 activity in the cell. Rheb is a membrane-bound protein (through its C-terminal prenylation) that is found predominantly in the Golgi ^67^; although a small fraction of Rheb plausibly becomes apposed with MTORC1 at the lysosome to initiate activation ^67^, MTORC1 thereafter operates in essentially every cellular compartment, including the nucleus ^68^. Fluorescence lifetime imaging of overexpressed Rheb, MTOR, and Raptor, each fused to fluorescent proteins, did detect the presence of a Rheb-MTOR complex in both the cytoplasm and the nucleus (the latter devoid of Raptor) ^69^; as yet, however, the presence of endogenous Rheb in the nucleus and its localization therein await confirmation. Moreover, Rheb membrane association suggests a specific orientation for MTORC1 position relative to the membrane, which would restrict cooperative activation only at the membrane. The mechanism by which MTORC1 activity is sustained within all cell compartments and the role of Rheb remain critical, unanswered questions.

## Conclusions

MTORC1 exists as an extremely elaborate large holoenzyme. Why go to all the trouble? The structure may highlight three important properties of MTORC1 functions:

1. Adaptor binding. The interface formed by the MTORC1 dimer provides a platform for Raptor. This same site is used by MTORC2 for RICTOR binding, building in a modularity often found in signaling assemblies.

2. Cooperative, allosteric activation. The mechanism for Rheb activation via HEAT/FAT domain re-organization is cooperative and is coupled through the dimeric interface mediated by the HEAT domains. The dimeric holoenzyme is required for the sigmoidal response. Could there be other modulators of MTORC1 activity that act through other allosteric sites?

3. Localization. The Rheb-dependent activation presumably occurs at a membrane surface. The location of the Rheb binding sites, if engaged simultaneously, would define a specific orientation of MTORC1 at the membrane. How this positioning influences its engagement with other upstream and downstream factors is one of the many unexplored questions that remain.
